# A myosin hypertrophic cardiomyopathy mutation disrupts the super-relaxed state and boosts contractility by enhanced actin attachment

**DOI:** 10.1073/pnas.2521561122

**Published:** 2025-12-24

**Authors:** Robert C. Cail, Bipasha Barua, Faviolla A. Báez-Cruz, Donald A. Winkelmann, Yale E. Goldman, E. Michael Ostap

**Affiliations:** ^a^Department of Physiology, Perelman School of Medicine, University of Pennsylvania, Philadelphia, PA 19104; ^b^Pennsylvania Muscle Institute, Perelman School of Medicine, University of Pennsylvania, Philadelphia, PA 19104; ^c^Department of Pathology and Laboratory Medicine, Robert Wood Johnson Medical School, Rutgers University, New Brunswick, NJ 07103; ^d^Department of Pharmacology, University of California Davis, Davis, CA 95616; ^e^Department of Molecular and Cell Biology, University of California Davis, Davis, CA 95616

**Keywords:** myosin, actomyosin, hypertrophic myopathy, optical trap, super-relaxed state

## Abstract

Hypertrophic cardiomyopathy (HCM) is a leading cause of cardiac failure among individuals under 35. Many genetic mutations that cause HCM seem to enhance ventricular systolic function, suggesting that these HCM mutations are hypercontractile. Among the most common causes of HCM are mutations in the gene MYH7, which encodes for β-cardiac myosin, the principal human ventricular myosin. Previous work has demonstrated that, for purified myosins, some MYH7 mutations are gain-of-function while others cause reduced function, so how they lead to enhanced contractility is not clear. Here, we have characterized the mechanics and kinetics of the severe HCM-causing mutation M493I. Motility assays demonstrate a 70% reduction of actin filament gliding velocities on M493I-coated surfaces relative to wild type (WT). This mutation slows ADP release from actomyosin•ADP fivefold without affecting phosphate release or ATP binding. Yet it enhances steady-state ATPase *V_max_* twofold. Through single-molecule mechanical studies, we find that M493I myosin has a normal working stroke of 5 nm but a significantly prolonged actin attachment duration. Under isometric feedback, M493I myosins produce high, sustained force, with an actin detachment rate that is less sensitive to force than that of WT myosin. We also report direct measurement of the equilibrium state of the super-relaxed to disordered relaxed regulatory transition and show its disruption in M493I, with a concomitant enhancement to actin attachment kinetics. Together, these data demonstrate that enhanced myosin binding from inhibition of myosin’s off state, combined with slow ADP release and enhanced force production, underlie the enhanced function and etiology of this HCM mutation.

## Significance

Hypertrophic cardiomyopathy (HCM) is a leading genetic cause of sudden cardiac death in young individuals. Although often described as a hypercontractile disease, the molecular basis for this remains unclear, especially for mutations with inhibitory effects in various in vitro assays. We show that the severe HCM mutation M493I in β-cardiac myosin slows ADP release yet enhances force output and actin attachment through multiple mechanisms, including disrupted autoinhibition via the super-relaxed state. Our findings unify seemingly contradictory biophysical changes into a coherent mechanistic model and support the hypothesis that increased myosin head availability, rather than enhanced individual kinetics alone, underlies HCM hypercontractility.

Hypertrophic cardiomyopathy (HCM) is an inherited genetic disorder that follows an autosomal dominant pattern and affects approximately 1 in 500 individuals (1). It is a leading cause of heart failure and sudden cardiac death, particularly in individuals under 35 (2). HCM is characterized by an abnormal thickening of the left ventricular free wall and intraventricular septum, along with cardiomyocyte disarray and interstitial fibrosis (3–6). These changes can lead to complications such as outflow obstruction, atrial fibrillation, heart failure, ventricular arrhythmias, and sudden cardiac death (1–8).

HCM-causing mutations can occur in approximately 20 sarcomeric genes, with mutations in the gene MYH7, encoding β-cardiac myosin, the principal paralog of myosin found in human ventricles, being implicated in 30 to 40% of cases (9, 10). The majority of these are classified as missense mutations (11). HCM is typically associated with impaired diastolic function while systolic function is either preserved or enhanced, leading to its classification as a hypercontractile disorder (12–14). However, mutations in β-cardiac myosin (hereafter, myosin) have been shown to either increase or decrease force production when analyzed in isolated myofibrils or single-molecule studies (15–19). How these opposing effects on myosin result in the same disease phenotype has been a matter of investigation and debate (16, 20, 21).

**Fig. 1.**
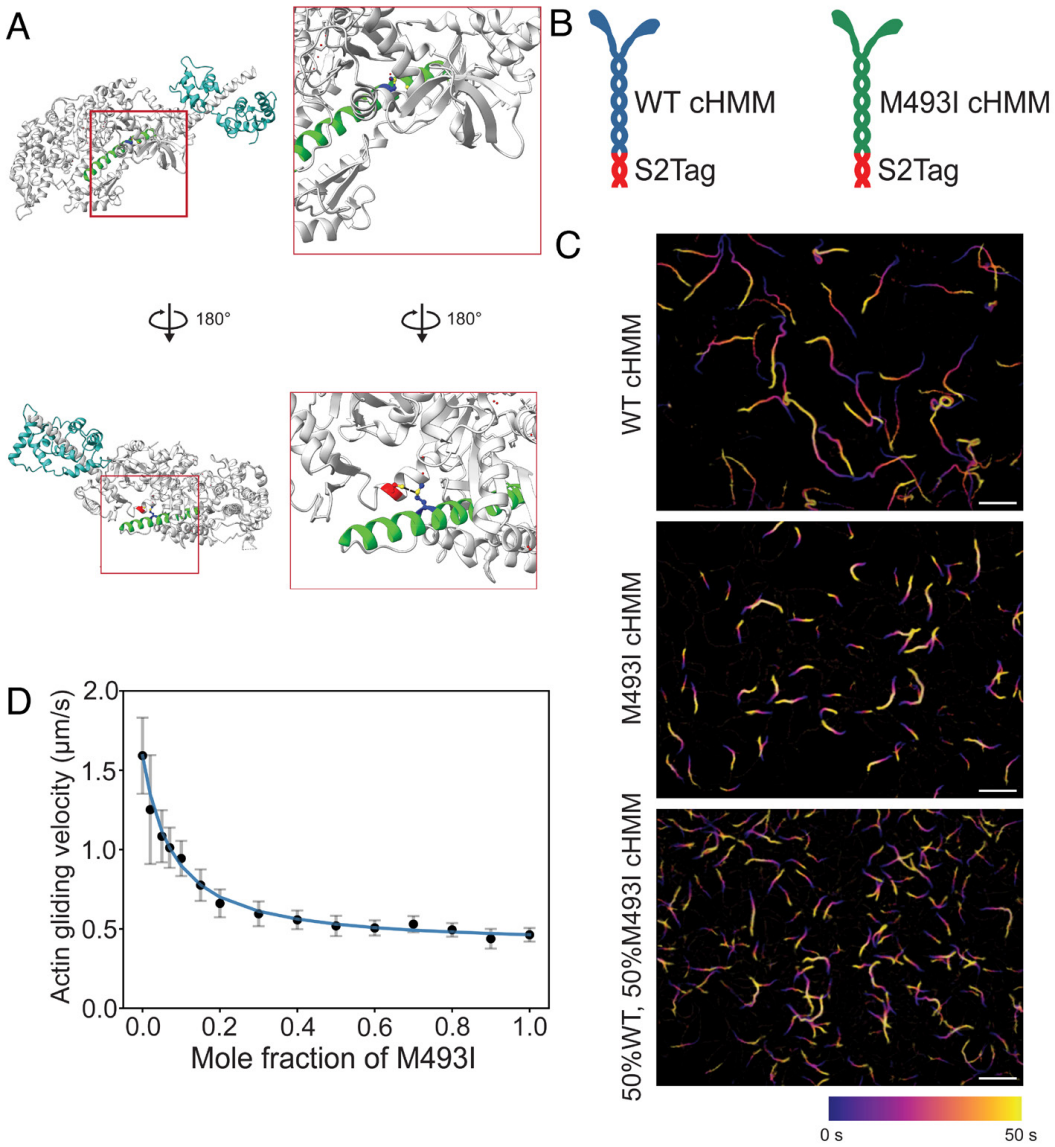
M493I is a relay helix mutation that significantly slows actin gliding velocity. (*A*) The relay helix (green) contains M493 (blue), which establishes a hydrogen bond with C705 (red) in the converter domain. (*B*) Schematic of purified WT- and M493I-cHMMs, containing 42 heptads of coiled-coil tail and engineered with a coiled-coil epitope S2Tag (10F12.3 subfragment of *G. domesticus* skeletal muscle myosin). (*C*) Color-coded time projections from actin gliding assays WT (*Top*), M493I (*Middle*), and 50-50 mixture of the two myosins (*Bottom*). Longer traces correspond to faster gliding velocity. (Scale bar: 10 μm.) (*D*) Actin gliding velocity as a function of mole fraction of M493I, demonstrating decreased gliding speeds with low proportions of M493I motors present.

One HCM-causing mutation, M493I, is linked to septal stiffening, severe physical limitations, congestive heart failure, and sudden cardiac death (22, 23). The M493 residue is located in the relay helix between the active site and the rotating converter domain of β-cardiac myosin, where it may form a hydrogen bond with C705 in the SH1 helix (24–28) (Fig. 1*A*). The relay helix plays a crucial role in transmitting the nucleotide state of the active site to the converter domain, coupling ATP hydrolysis and product release to the tilting of myosin’s lever arm (29, 30). It is highly conserved across myosin paralogs (*SI Appendix*, Fig. S1*A*) (31). While M493 is conserved across fast class II myosins in humans, other myosin paralogs have different amino acids in this position, such as isoleucine (class VI and IX myosins) or valine (class V and VII myosins). The region near M493 is a hotspot for HCM- and dilated cardiomyopathy (DCM)-causing mutations, including residues E497, Y501, and F513 in the relay helix, as well as R712 and F764 in the converter domain (16, 25–28).

In addition to the canonical actin-activated ATPase mechanism (32), many class II myosins, including β-cardiac myosin, have a conserved “off” state that prevents actin binding (33–35). This state is stabilized by an interaction between the motor domain (head) and the dimer- and filament-forming tail domain, and it is regulated by phosphorylation and other factors. In muscle myosins, this state is referred to as the interacting-head motif (IHM) and is commonly linked to the biochemically defined super-relaxed (SRX) state of myosin (36–38), with FRET structural studies finding good correlation between the IHM and SRX state (39, 40). The SRX state is thought to consume ATP at a 10-fold slower rate than the alternative disordered relaxed (DRX) state, in which the myosin heads are more available for actin binding. The mechanisms and kinetics governing the SRX/DRX transition for purified heavy meromyosin (HMM) remain unclear. However, structural studies have shown that the IHM state corresponds to the pre-power-stroke (PPS) conformation, with ADP•P_i_ bound in the active site (41).

Efforts to develop a unifying model of HCM have led to the “head availability hypothesis” (14, 20, 42–44). This hypothesis suggests that HCM-causing mutations disrupt the SRX/DRX balance, increasing the number of myosin heads in the DRX (available) state. This increase in available heads causes excessive activation of the thin filament (TF) and hypercontractility, regardless of the mutation’s direct effects on intrinsic myosin kinetics, working stroke size, and power output. A recent review found through an assay comparing different constructs, termed the long-tail/short-tail ATPase ratio, that HCM-causing mutations generally, but not universally, reduce the inhibitory effect of the proximal S2 tail on myosin’s actin-activated ATPase activity, thereby increasing the proportion of available heads (20). The effects of most HCM-causing mutations on SRX/DRX balance have not been directly measured.

In this study, we characterized the impact of the M493I mutation on the mechanochemistry of a recombinant HMM construct. We applied ensemble kinetics and the single-molecule optical trapping technique to measure the effect of the mutation on key ATPase cycle transitions, linking these changes to specific substeps of energy transduction by myosin. Our findings provide evidence that the SRX/DRX equilibrium is fast relative to ATP turnover, along with an estimation of its equilibrium constant, insights into how the M493I mutation alters head availability in myocytes, and a measurement of the enhanced actin-binding activity at the single-molecule level induced by M493I. Together, these results point to a mechanism by which a single HCM-linked point mutation alters myosin’s mechanochemical cycle to promote hypercontractility.

## Results

### M493I Slows Actin Gliding Velocity.

Human β-cardiac myosin wild type HMM (WT-cHMM) and M493I mutant (M493I-cHMM) constructs, with 42 heptads of the coiled-coil S2 tail domain, were expressed in mouse C2C12 myoblasts and purified (*SI Appendix*, Fig. S1*B*) (16, 27). At the C-terminus of the S2 domain, the S2Tag (a coiled-coil epitope tag composed of the 10F12.3 region of *Gallus domesticus* skeletal muscle myosin) was included, allowing for site-specific binding of purified myosins through a monoclonal antibody to this epitope tag (Fig. 1*B*) (45).

To assess whether the M493I mutation affects unloaded actin motility, we conducted an in vitro actin gliding assay using both WT and M493I myosins. Adhering the myosin constructs via an antibody to the S2Tag enabled precise deposition of mixed mole fractions of WT and M493I myosins onto the coverslips at a total loading concentration of 10 µg/mL. WT myosin exhibited persistent actin propulsion, with a velocity of 1.6 ± 0.2 µm/s (Fig. 1 *C* and *D*, Table 1, and Movie S1). M493I myosin also supported smooth continuous actin gliding but at a 72% slower velocity (0.46 ± 0.04 µm/s; Fig. 1 *C* and *D*, Table 1, and Movie S2). When WT and M493I myosins were co-incubated in varying proportions, actin gliding velocity declined sharply with increasing M493I mole fraction, showing a 50% velocity reduction at just 10% proportion of mutant myosin and approaching the low-velocity asymptote of the velocity curve at ~50% M493I myosin (Fig. 1 *C* and *D*, Table 1, and Movie S3). These data were fitted to a quadratic model of loaded force production in the presence of mixed myosin species, as described previously (*Materials and Methods*) (46, 47). The concave shape of this curve suggests that the faster WT-cHMM experiences frictional loading by the slower M493I-cHMM, indicative of drag due to enhanced actin attachment of the mutant version (47). To further investigate this frictional slowing, we examined which steps of the mechanochemical cycle were affected by the M493I mutation.

**Table 1.** Kinetic and mechanical parameters of WT-cHMM and M493I-cHMM

	WT-cHMM	M493I-cHMM	*P* (Welch’s *t* test)	Method
Actin gliding velocity (µm/s)	1.6 ± 0.2	0.46 ± 0.04	*P* < 0.0001	In vitro actin gliding velocity with rhodamine-phalloidin labeled actin
P_i_ release rate (fast phase) (s^−1^)	15.8 ± 5.0	15.1 ± 4.0	NS (*P* = 0.07)	Phosphate-binding protein fluorescence, SF, 30 μM porcine TFs, TF-calcium buffer
P_i_ release fast, fractional amplitude	0.27	1	N/A	Phosphate-binding protein fluorescence, SF, 30 μM porcine TFs, TF-calcium buffer
P_i_ release rate (slow phase) (s^−1^)	0.37 ± 0.08	N/A	N/A	Phosphate-binding protein fluorescence, SF, 30 μM porcine TFs, TF-calcium buffer
ADP release rate (s^−1^)	69 ± 7	12.0 ± 0.8	*P* < 0.0001	Pyrene-actin, SF
ATP binding second-order rate (µM^−1^ s^−1^)	4.7 (4.6-5.2, 95% CI)	6.2 (5.8-6.4, 95% CI)	*P* < 0.0001	Pyrene-actin, SF
Maximum rate ATP-induced dissociation of actomyosin	1,191 ± 109^*^ 941 ± 60^†^	340 (322-419, 95% CI)	*P* < 0.0001	Pyrene-actin, SF
ATP binding *K*_M_ (μM)	128 ± 29^†^	35.4 (24.7-61.8, 95% CI)	*P* < 0.0001	Pyrene-actin, SF
Steady-state ATPase *Vmax* (s^−1^)	1.20 (1.09-1.77, 95% CI)	2.35 (1.51-2.51, 95% CI)	*P* = 0.0002	NADH-coupled ATPase assay, spectrophotometer, porcine TFs, TF-calcium buffer
Steady-state ATPase *K_M_*(µM)	6.66 (5.25-13.62, 95% CI)	7.65 (2.97-10.09, 95% CI)	NS (*P* = 0.72)	NADH-coupled ATPase assay, spectrophotometer, porcine TFs, TF-calcium buffer
*k_detach_* (1 μM ATP) (s^−1^)	6.09 (5.66-6.59, 95% CI)	6.26 (5.89-6.68, 95% CI)	NS (*P* = 0.59)	3-bead optical trap assay
*k_detach_* (2 mM ATP) (s^−1^)	58.48 (51.95-67.02, 95% CI)	12.06 (11.11-13.26, 95% CI)	*P* < 0.0001	3-bead optical trap assay
Substep 1 (nm)	3.88 ± 0.23 (SEM)	3.80 ± 0.22 (SEM)	NS (*P* = 0.80)	3-bead optical trap assay
Substep 2 (nm)	0.94 ± 0.18 (SEM)	0.88 ± 0.15 (SEM)	NS (*P* = 0.80)	3-bead optical trap assay
Total step size (nm)	4.82 ± 0.25 (SEM)	4.68 ± 0.21 (SEM)	NS (*P* = 0.67)	3-bead optical trap assay
Bell equation *k_0_* (s^−1^)	32.9 (28.8-35.3, 95% CI)	12.9 (9.2-18.2, 95% CI)	*P* < 0.0001	3-bead optical trap assay, isometric FB
Bell equation distance parameter (nm)	0.80 (0.62-0.97, 95% CI)	0.32 (0.00-0.70, 95% CI)	*P* = 0.016	3-bead optical trap assay, isometric FB
Single-nucleotide turnover rate (s^−1^)	0.0047 ± 0.0005^‡^	0.009 ± 0.001	*P* = 0.0004	mantATP turnover, SF
Single-nucleotide turnover rate (undigested, in papain digestion buffer) (s^−1^)	0.0042 ± 0.0004^‡^	0.0065 ± 0.0003	*P* = 0.002	mantATP turnover, SF
Single-nucleotide turnover rate (papain-digested in papain digestion buffer) (s^−1^)	0.013 ± 0.001^‡^	0.017 ± 0.002	*P* = 0.012	mantATP turnover, SF
SRX-DRX equilibrium constant	0.32 ± 0.03^‡^	0.53 ± 0.03	*P* = 0.013	mantATP turnover, SF
Duty Ratio	0.017	0.2	N/A	Calculated from measured values
Actin reattachment rate in optical trap (slow) (s^−1^)	0.83 (0.70-0.9, 95% CI)	1.62 (1.45-1.74, 95% CI)	*P* < 0.0001	3-bead optical trap assay, pedestal bead stage feedback
Actin reattachment slow amplitude	80% (65-87, 95% CI)	81% (71-85, 95% CI)	NS (*P* = 0.88)	3-bead optical trap assay, pedestal bead stage feedback
Actin reattachment rate in optical trap (fast) (s^−1^)	17 (5.5-36, 95% CI)	23 (11-39, 95% CI)	NS (*P* = 0.57)	3-bead optical trap assay, pedestal bead stage feedback

Experiments performed in KMg25 buffer unless otherwise noted.

^*^Published previously (16).

^†^Published previously (45).

^‡^Published previously (48).

### M493I Preserves P_i_ Release and ATP Binding but Slows ADP Release.

We investigated whether the M493I mutation alters key rate constants in the ATPase cycle that regulate entry into and exit from the force-bearing, actin-bound states using stopped-flow kinetics (*SI Appendix*, Fig. S1*C*, Scheme 1) (49). The rate of the phosphate release step, which limits entry into the strong-binding states of the ATPase cycle, was measured by fluorescent Pi binding protein (50, 51) in the presence of a saturating concentration (30 µM subunits) of porcine ventricular TFs (*SI Appendix*, Fig. S2*A*). As found previously (51, 52), P_i_ release transients in the presence of WT-cHMM were best fit by a two-exponential function, with a fast-phase (15.8 ± 5 s^−1^, 27% of the transient amplitude, *SI Appendix*, Fig. S2*B* and Table 1) and slow-phase (0.37 ± 0.08 s^−1^, 73% of the transient amplitude, *SI Appendix*, Fig. S2*B* and Table 1). M493I-cHMM released Pi in a single exponential phase at a rate of 15.1 ± 4 s^−1^ (*SI Appendix*, Fig. S2*C* and Table 1), with a total amplitude that was equivalent to the total amplitude of the WT-cHMM. This rate is statistically indistinguishable from the fast-phase rate for WT-cHMM, which is thought to be the actin-activated Pi release rate in the myocardium. The slow phase of phosphate release is present in both cHMM and cS1 myosin preparations (51–53). The absence of a slow phase for M493I-cHMM may indicate that a secondary phosphate release pathway, present in WT-cHMM, is absent or undetectable with the M493I mutation.

To evaluate exit from the force-bearing states (*SI Appendix*, Fig. S1*C*, Scheme 1), we measured the rate of ADP release and ATP binding to actin-attached myosin bound by dissociation from pyrene-actin (*Materials and Methods* and *SI Appendix*, Fig. S2 *D* and *E*). ADP release from M493I-cHMM was approximately fivefold slower (12.0 ± 0.8 s^−1^; Table 1) than from WT-cHMM (69 ± 7 s^−1^; Table 1). Slowed ADP release from actomyosin may be caused by increased affinity of M493I-cHMM for ADP. ATP-induced dissociation of pyrene-actin-bound myosin was measured for a range of MgATP concentrations (500 nM to 0.6 mM, *SI Appendix*, Fig. S1*C*, Scheme 1). Pyrene fluorescence transients were well fit by single exponential functions (*SI Appendix*, Fig. S2 *F* and *G*). From the ATP dependence of the observed rates of ATP-induced actomyosin dissociation at low [ATP] (500 nM to 10 µM), the apparent second-order rate constant for ATP binding to M493I-cHMM [6.2 (µM s)^−1^; 5.8 to 6.4 (µM s)^−1^ 95% CI] was determined to be slightly faster than WT-cHMM [4.7 (µM s)^−1^; 4.6 to 5.2 (µM s)^−1^ 95% CI; *SI Appendix*, Fig. S2*H* and Table 1]. However, the maximum rate of ATP-induced dissociation for M493I-cHMM (340 s^−1^; 322 to 419 s^−1^ 95% CI) was slower than the previously determined rate for WT-cHMM (1,191 ± 109 s^−1^) (*SI Appendix*, Fig. S2*I*) (16). The maximum rate of ATP-induced actomyosin dissociation is reduced 3.5-fold in M493I-cHMM, and the Km is similarly reduced for M493I-cHMM (35.4 µM; 24.7 to 61.8 µM 95% CI) relative to WT-cHMM (128 ± 29 µM), and these effects result in a similar second-order rate constant for ATP binding. ADP release rather than ATP binding limits exit from the strong-binding states under mM physiological ATP concentrations. Thus, these findings suggest that the observed slowing of actin gliding is primarily attributable to the reduced rate of ADP release.

### M493I Preserves the Power Stroke and Enhances Actin Attachment Duration.

Some HCM-causing mutations in myosin can significantly alter the power stroke and actin filament sliding, particularly those near the lever arm (16, 17, 19). We examined the impact of the M493I mutation on myosin’s power stroke using optical trapping assays to assess how this relay-helix mutation affects motor properties, providing insights into its potential implications for muscle physiology.

**Fig. 2.**
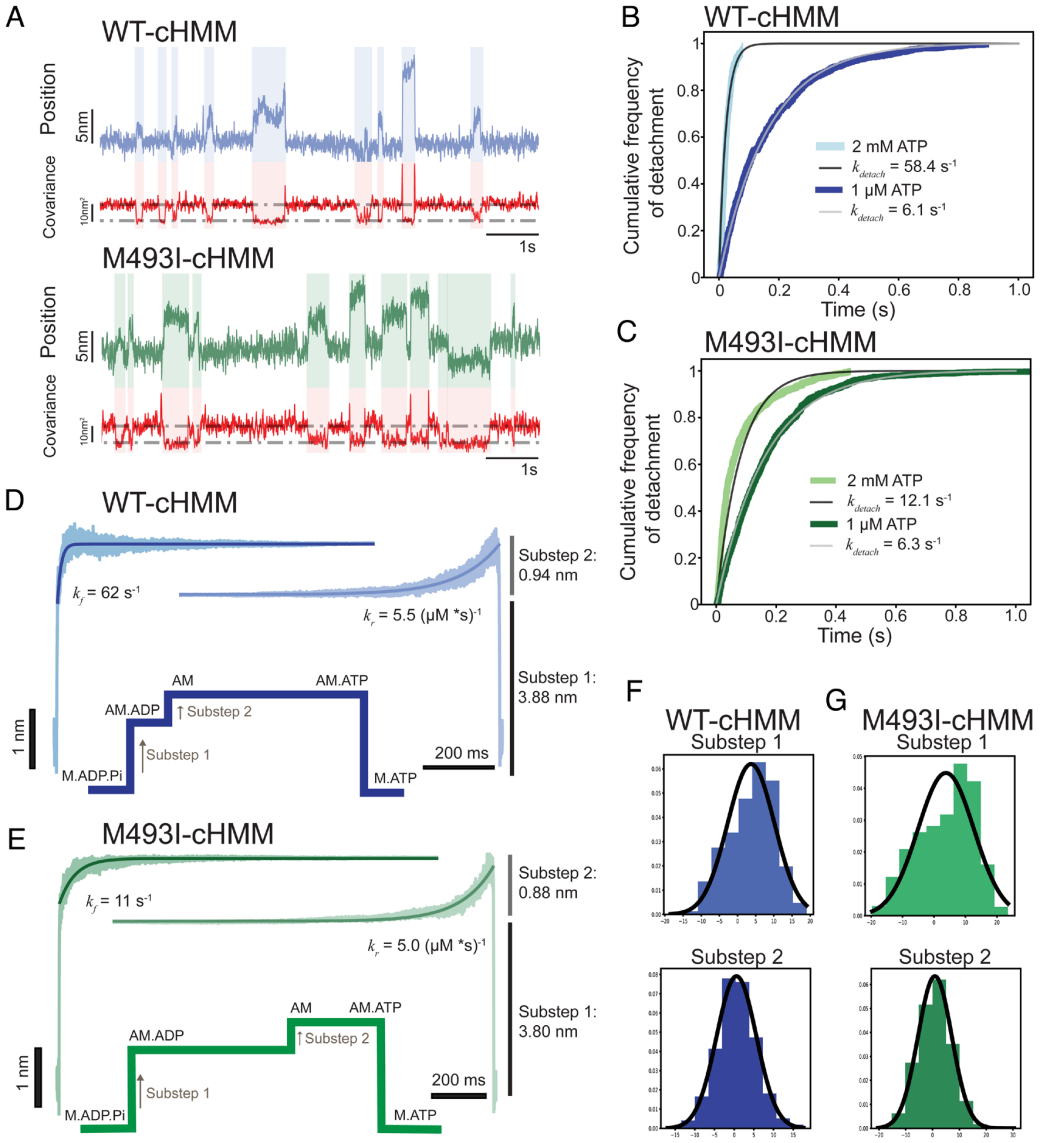
Step size and duration for WT and M493I myosins. (*A*) Sample traces from unloaded optical trap assays of WT and M493I-cHMM. (*B* and *C*) Cumulative distributions of attachment durations with fitted single-exponential curves showing the difference in attachment lifetime at high [ATP] between WT (*B*) and M493I-myosin (*C*). (*D* and *E*) Ensemble averages and single-exponential fits of WT (*D*) and M493I (*E*) myosin events at 1 μM [ATP] demonstrating the same step amplitudes but slowed transition from substep 1 to 2 for M493I myosin. (*F* and *G*) Average substep sizes for WT (*F*) and M493I (*G*) myosins with fitted Gaussian curves.

Pedestal beads, sparsely coated with antibodies against the S2Tag, were used to bind WT-cHMM or M493I-cHMM, at sufficiently sparse coverage (with only one in five beads interacting with actin) to achieve single molecule events. An actin filament was suspended between two optically trapped beads, and bead position and variance were monitored. Single actomyosin binding events were detected by a decrease in covariance between the beads (Fig. 2*A*) (54, 55). For both WT and M493I myosins at 1 µM MgATP, transient decreases in covariance corresponded with bead displacement during attachment events (Fig. 2*A*). Interaction duration decreased with increasing ATP, following single-exponential cumulative distribution. The detachment rate (*k*_detach_) at 1 µM ATP was 6.09 s^−1^ (5.66 to 6.59 s^−1^ 95% CI) for WT and 6.26 s^−1^ (5.89 to 6.68 s^−1^ 95% CI) for M493I, consistent with detachment rate being limited by ATP binding (Fig. 2 *B* and *C*). At 2 mM MgATP, actin detachment rate is limited by ADP release and M493I detached more slowly (*k*detach = 12.06 s^−1^, 11.11 to 13.26 s^−1^ 95% CI) compared to WT (*k*detach = 58.48 s^−1^, 51.95 to 67.02 s^−1^ 95% CI). This result demonstrates that the M493I mutation increases the duration of myosin attachments to actin, consistent with slowed ADP release which, in turn, explains its slowing of in vitro filament gliding (Fig. 2 *B* and *C*).

The cardiac myosin power stroke exhibits two barbed-end directed steps, linked to 1) P_i_ release from AM•ADP•P_i_, and 2) ADP release from AM•ADP. The average total amplitude of the working stroke was determined by combining single-molecule interactions aligned at initial attachment times (time-forward ensemble averages) and detachment times (time-reversed averages) (Fig. 2 *D* and *E*) (56, 57). For time-forward averages, detected events were aligned at the start of the interaction, extending shorter events for averaging in the software to same duration as longest detected interaction for averaging. The exponential increase in these averages after the initial displacement is caused by the transition from substep 1 to substep 2, i.e., ADP release (55–57). For reverse averages, the ends of each interaction were aligned, extending each shorter interaction back in time to match the longest duration for averaging. The exponential increase in displacement in these averages reports the rate of ATP binding before detachment.

For wild-type myosin, substeps 1 and 2 had displacements of 3.88 ± 0.23 nm (SEM) and 0.94 ± 0.18 nm (SEM) for a total step size of 4.82 ± 0.25 nm (SEM) (Fig. 2 *D* and *F*). M493I-myosin’s ensemble averages a 1 µM ATP revealed a similar two-substep working stroke as that of WT-myosin with substep 1 and 2 displacements of 3.80 ± 0.22 nm (SEM) and 0.88 ± 0.15 nm (SEM) for a similar total step size of 4.68 ± 0.21 nm (SEM) (Fig. 2 *D* and *G*).

For WT-myosin at 1 µM ATP, the ADP release rate and ATP binding rate determined by fitting the exponential phases of the forward and reverse ensemble averages, respectively, were 62 s^−1^ (58 to 71, 95% CI) for the transition from substep 1 to substep 2 and 5.5 s^−1^ (3.6 to 8.6, 95% CI) for ATP-dependent detachment, in good agreement with values from pyrene-actin bulk transients (Fig. 2*D*). For M493I-myosin, the rate for transition from substep 1 to substep 2 was 11 s^−1^ (9.5 to 13.3, 95% CI) similar to the 12.0 s^−1^ ADP off-rate as measured from the bulk pyrene actin kinetics, but slower than WT. The rate constant for detachment due to ATP binding was 5.0 s^−1^ (4.2 to 6.5, 95% CI) similar to WT and to the 6.2 s^−1^ at 1 µM ATP measured by pyrene actin (Fig. 2*E*). Thus, M493I alters the working stroke of actomyosin not by altering the step size or ATP binding rate, but by slowing detachment through slowing the transition from substep 1 to substep 2, consistent with slowed ADP release and enhanced frictional loading of a moving actin filament.

### Under Isometric Tension, M493I Produces Remarkably Long-Duration, High-Force Actin Attachments.

Myosin’s loaded force generation capability, with associated load-dependent release of ADP release, is a key factor in effective, synchronized cardiac muscle shortening and energetic regulation (55, 58, 59,60, 61). HCM-causing mutations in MYH7 can either increase or decrease the force produced by actomyosin interactions (16, 17, 19). We sought to investigate the effect of the mutation M493I on force production and load-dependent ADP dissociation kinetics using the three-bead optical trap assay under isometric feedback conditions (62). In this assay, the position of the bead at the pointed end of the actin dumbbell (termed the transducer bead) is monitored optically, and the position of the barbed-end bead (the motor bead) is adjusted by feedback to an electro-optic deflector to minimize (clamp) the displacement of the transducer bead, thus approximating the isometric condition in a muscle independent of series compliances in the dumbbell. When myosin interacts with the actin filament, it is subjected to hindering load applied by this feedback as it produces its working stroke, and we can assay both its force generation capability and its loaded ADP dissociation rate.

**Fig. 3.**
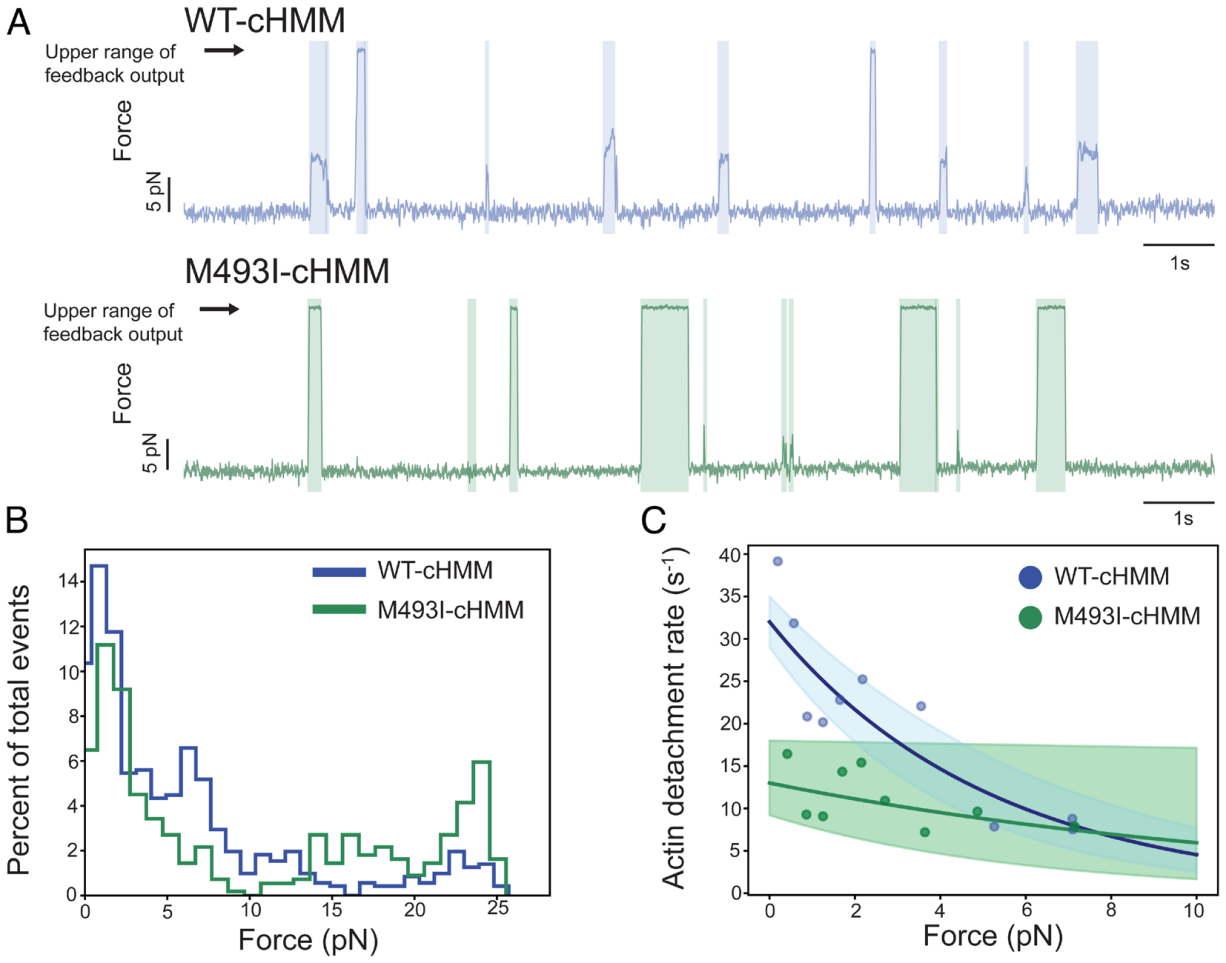
Force production by WT and M493I myosins. (*A*) Sample traces from isometric feedback optical trap experiments showing a high proportion of feedback-saturating events present with M493I myosin. (*B*) Histograms of force distribution for WT and M493I myosins. The mutant exhibits more high-force (>10 pN) events than WT. (*C*) Actin detachment rate vs. force for WT (blue) and M493I (green) myosins showing similar force-induced slowing of ADP release. Data are averaged in 25-point bins. Solid lines represent fits to the Bell equation applied to unbinned data, and shaded areas indicate the 95% CI, derived from bootstrap-based Bell equation fits (*Materials and Methods*).

WT-cHMM demonstrates regular interactions of variable force under load, as expected from the thermal (Brownian) fluctuations at the moment of attachment (Fig. 3 *A*, *Top*) (55, 59). M493I-cHMM interactions under load are striking in their duration and force magnitude to a level that often exceeds the 25 pN dynamic range of our isometric clamp feedback loop (Fig. 3 *A*, *Bottom*). Histograms of the forces from individual interactions demonstrate a considerable increase in the proportion of high-force interactions for M493I myosins relative to WT; the force measurements plotted are an underestimate because the highest force value is limited by the aforementioned dynamic range of the instrument (Fig. 3*B*). We propose that these high forces are the result of recruitment of the second head of the HMM.

When interactions >10 pN are excluded from both datasets (to exclude possible second-head interactions and limit the distributions to interactions within the subsaturating force range of the feedback loop), the detachment rate vs. force data can be fitted to the Bell equation (63),

[1] $$k_{\mathrm{obs}}=k_{0}*e^{-\frac{F*d}{k_{B}*T}},$$

where *k_obs_* is the observed detachment rate under load, *k*_0_ is the detachment rate (limited by ADP release) at zero force, *F* is the force applied to the myosin, k_B_*T is the Boltzmann constant times temperature, and *d* is the distance parameter, the effective displacement between the force-generating attached position and the transition barrier to detachment. We expect that for individual motor domains, the predicted rates will follow the Bell model when using the distance parameter determined from interactions below 10 pN. In contrast, the two-headed construct exhibits lifetimes that deviate from Bell-like behavior, indicating additional force-dependent processes, likely due to two-headed binding, that are not captured by the simple model. WT *k_0_* = 32.0 s^−1^ (28.8 to 35.3 s^−1^ 95% CI) and *d* = 0.80 nm (0.62 to 0.97 nm 95% CI), whereas for M493I, *k_0_* = 12.9 s^−1^ (9.2 to 18.2 s^−1^ 95% CI) and *d* = 0.32 nm (0.00 to 0.70 nm 95% CI) (Fig. 3*C*). The reduction in distance parameter indicates that the ADP release rate of M493I-cHMM is less sensitive to force than WT-cHMM, a possible contributing factor to asynchronous contraction in heterozygous disease myocardium. These effects on force production and ADP release may contribute to gain of function, which leads to septal hypertrophy and ultimately outflow restriction present in M493I patients (22, 23).

### M493I Enhances Steady-State ATPase Rates.

The actin-activated steady-state ATPase rate was measured using a NADH-coupled ATPase assay, over a range of concentrations of porcine cardiac TFs (*SI Appendix*, Fig. S1*C*, Scheme 1) (49). NADH absorbance decreases linearly over time with the slope indicating ATPase rate increasing with TF concentration (*SI Appendix*, Fig. S3 *A* and *B*). M493I myosins demonstrated higher ATPase rate than WT (*SI Appendix*, Fig. S3 *A*–*C*). The *V_max_* of the per-head ATPase rate for WT-cHMM was 1.20 s^−1^ (1.09 to 1.77 s^−1^ 95% CI) while for M493I-cHMM it nearly doubled to 2.35 s^−1^ (1.51 to 2.51 s^−1^ 95% CI) without significantly altering the *K*m (6.66 µM, 5.25 to 13.62 µM 95% CI for WT vs. 7.65 µM, 2.97 to 10.09 µM 95% CI for M493I). Thus, the slowed in vitro gliding filament motility is not caused by defective ATP cycling, as this rate is enhanced by the mutation (*SI Appendix*, Fig. S3*C* and Table 1).

### Absent Actin, M493I Enhances ATP Turnover and Reduces the Equilibrium Population of SRX Myosin Heads.

Enhancement of *V_max_* for M493I-cHMM steady-state ATPase seems in conflict with the slowed ADP release observed from pyrene and optical trapping experiments, which should tend to slow cycling through the ATPase pathway. A possible explanation for this discrepancy would be a difference in the number of available catalytic myosin heads. Class II myosins from across animalia have a conserved off state, in which the two heads of myosin fold onto the proximal coiled-coil tail, forming stabilizing interactions with both the other head and the tail domain (*SI Appendix*, Fig. S1*C*, Scheme 1) (33). In striated muscle myosins, this state is termed the IHM, and it has been correlated with a biochemically defined off state termed the SRX state (34, 36,43, 44, 64, 65). In contrast, myosin heads in the alternative disordered-relaxed (DRX) state are available to interact with the TF.

A common method for measuring the SRX/DRX partition is through single-turnover studies using a fluorescent ATP analog, N-methylanthraniloyl adenosine 5’-triphosphate (mantATP). In this approach, the proportion of fast and slow release of mantADP, either with purified myosin constructs or in isolated myofibrils/myocytes is estimated by fitting the fluorescence change upon mantADP dissociation, as a proxy for nucleotide turnover, to a double exponential model (34, 36–38). For purified HMM, the presence of two-phase nucleotide release has been interpreted as indicating that the transition out of SRX is rate-limiting (40,41, 42, 64). Recent work from our group and others has found instead that nucleotide release occurs in a single phase, implying a rapid equilibrium between the SRX and DRX states (48, 66). While earlier work from our group found that recombinant WT-cS1 released nucleotide more rapidly than WT-cHMM, other groups see no difference in purified cS1 and cHMM (66); these differences may be due to different methods for purifying protein or different conditions such as salt concentration.

**Fig. 4.**
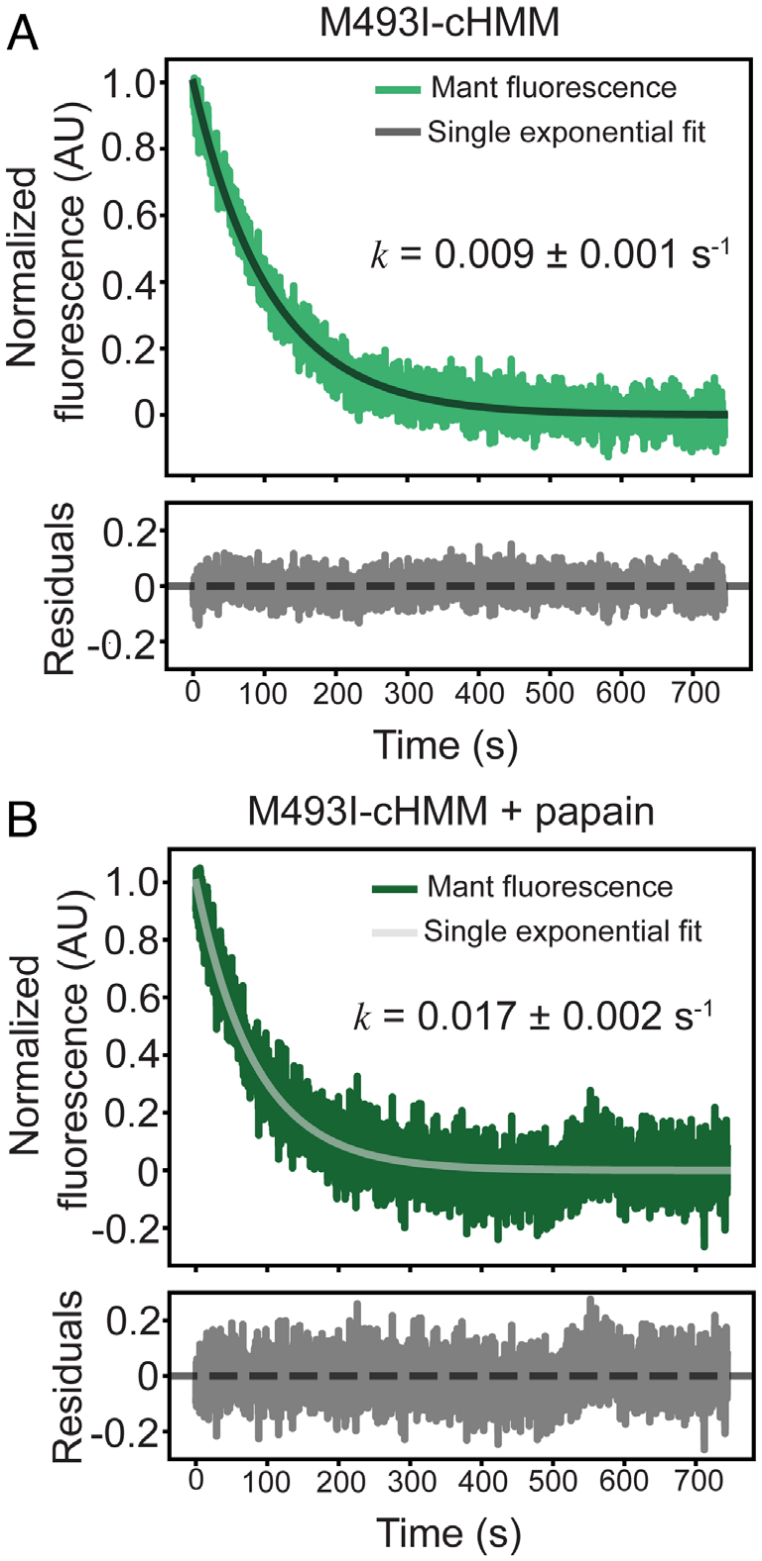
Turnover of MantATP for WT and M493I myosins. (*A*) M493I-cHMM MantADP release with single exponential fit. (*B*) MantADP release rate for M493I-cHMM after papain digestion, showing increased dissociation rate of release attributed to minimized (or lack of) population of SRX.

To assess the effect of the M493I mutation on the partitioning between the SRX and DRX states, we performed single-nucleotide turnover experiments using mantATP (Fig. 4*A*). In this assay, purified cHMM was rapidly mixed with a slight excess (1.1-fold) of mantATP, aged for 10 s to allow nucleotide binding and hydrolysis, and then chased with saturating unlabeled ATP (1 mM). The high ATP concentration ensures that, promptly after the 2nd mixing step, all the myosin heads have nucleotide bound and that the fluorescent nucleotide is unlikely to rebind to a myosin after dissociation. Tryptophan 508 in the active site was excited at 295 nm, resulting in fluorescence resonance energy transfer (FRET) excitation of the bound mant fluorophore. Upon nucleotide dissociation, fluorescence intensity decreased, and the decay in signal was fit to a single-exponential function corresponding to the basal rate of nucleotide release. A trace of mantATP fluorescence alone in KMg25 buffer, collected during the same type of experiment (48), demonstrates significant photobleaching of the mantATP, approximately 10% of the amplitude of the trace with purified myosin; this recording is subtracted from all of the myosin traces collected to correct for the photobleaching during acquisition.

In experiments with soluble cardiac myosin, we found that correction for photobleaching eliminated the previously reported slower phase attributed to a slow SRX-to-DRX transition. Instead, the presence of the S2 tail establishes a dynamic equilibrium between SRX and DRX that is faster than the rate of nucleotide turnover itself, as summarized by the following scheme:


$$\begin{array}{c} \text{SRX-M}{\text{.ADP}}^{**}.P_{i}{⇄}_{k_{-1}}^{k_{+1}}\text{DRX-M}{\text{.ADP}}^{**}.P_{i}\overset{k_{\text{DRX}}}{\to }\\ \;\text{M}+P_{i}+.{\text{ADP}}^{*} \end{array}$$

where M is myosin, ADP** is the high-fluorescence mantADP in the active site and ADP* is the low-fluorescence mantADP in solution (48). P_i_ release in DRX heads is rate-limiting for the measured *k_DRX_*. The equilibrium constant was determined according to:

[2] $$k_{\mathrm{obs}}=k_{\mathrm{DRX}}\cdot [DRX]/[SRX]=k_{\mathrm{DRX}}\cdot (k_{+1}/k_{-1})=k_{\mathrm{DRX}}\cdot K_{\mathrm{EQ}},$$

where *k_DRX_* is the elementary product release rate measured from cS1*, k*_+1_, and *k*_-1_ are the interconversion rates between SRX and DRX, and *K_EQ_ =* [DRX]/[SRX] = *k*_+1_/*k*_-1_.

As determined previously, WT-cHMM releases nucleotide in a single phase at a rate of 0.0047 ± 0.0005 s^−1^ (48). WT-subfragment 1, either recombinantly expressed or produced by limited papain digestion which cleaves HMM into S1, S2, and single headed fragments (48), releases nucleotide at the significantly increased rate of 0.013 ± 0.001 s^−1^, from which we calculate an equilibrium constant *K*SRX/DRX = 0.33 ± 0.05.

M493I-cHMM releases nucleotide with single-exponential kinetics, with a rate of 0.009 ± 0.001 s^−1^, approximately double that of WT-cHMM (Fig. 4*A*). Upon papain digestion, the nucleotide release rate again increased from 0.0065 ± 0.0003 to 0.017 ± 0.002 s^−1^, quite similar to the value for papain-digested WT-cHMM (Fig. 4*B* and *SI Appendix*, Fig. S4*A*). Paired undigested M493I-cHMM control samples again displayed a similar rate constant to untreated samples (*SI Appendix*, Fig. S4*B*). Thus, we estimate that M493I-cHMM is in equilibrium between SRX and DRX at a rate substantially faster than the ATP turnover rate with an equilibrium constant *K*SRX/DRX = 0.53 ± 0.03, a 60% increase over WT-cHMM. The higher availability of DRX heads actively hydrolyzing ATP provides an explanation for the higher steady-state ATPase rate of the mutant myosin despite the slower ADP release.

We estimate the duty ratio (*DR*) of the motor domains in cHMM as

[3] $$DR=\frac{timeinstrongbindingstate}{totalcycletime}=\frac{1/k_{\mathrm{ADP}}}{1/V_{\max}},$$

assuming 1/*V_max_* represents the cycle time of the motor, and that the AM.ADP state is the predominant strong-binding state with 1/*k_ADP_* representing its lifetime. It is important to note that in this calculation, the time myosin spends in the SRX state influences the *V_max_*; when turnover rate of the whole myosin population (SRX and DRX) is considered. Thus, an increase in SRX occupancy decreases the DR and ultimately reduces the total power generated by the cHMM molecules. The *DR* of WT-cHMM in the absence of force is 0.017, consistent with previously measured values, while M493I-cHMM exhibits a >10-fold higher unloaded *DR* = 0.20 (17, 19). The measured equilibrium constants for SRX-DRX transition imply that 25% of WT-cHMM heads are in the DRX state, while for M493I this proportion increases to 35%. With these values, we estimate the *DR* = 0.069 for WT-cS1, which does not populate the SRX state. Strikingly, the *DR* = 0.56 for M493I-cS1, which is approaching the values seen in transport myosin classes (67).

The force-dependent power [*P*(*F*)] of WT-cHMM and M493I-cHMM was calculated according to the following equation (68):

[4] $$P\left(F\right)=F*d*k_{\mathrm{ADP}}(F),$$

where *k*_ADP_(*F*) is the force-dependent rate that limits the speed of shortening (Fig. 3*C*) and *d* is the actin displacement produced by the myosin. Without accounting for differences in DR, the estimated power output of WT-cHMM appears substantially greater than that of M493I-cHMM, owing to its higher ADP release rate (*SI Appendix*, Fig. S4 *C*, *Inset*). However, after correcting for the higher DR of M493I-cHMM (Eq. **4** * Eq. **3**, where *k_ADP_* is force-dependent in both equations), we find that the power generated by M493I-cHMM is nearly twofold higher than that of WT-cHMM over the 0 to 10 pN force range (*SI Appendix*, Fig. S4*C*).

### M493I Enhances Single-Molecule Actin Rebinding Rate.

**Fig. 5.**
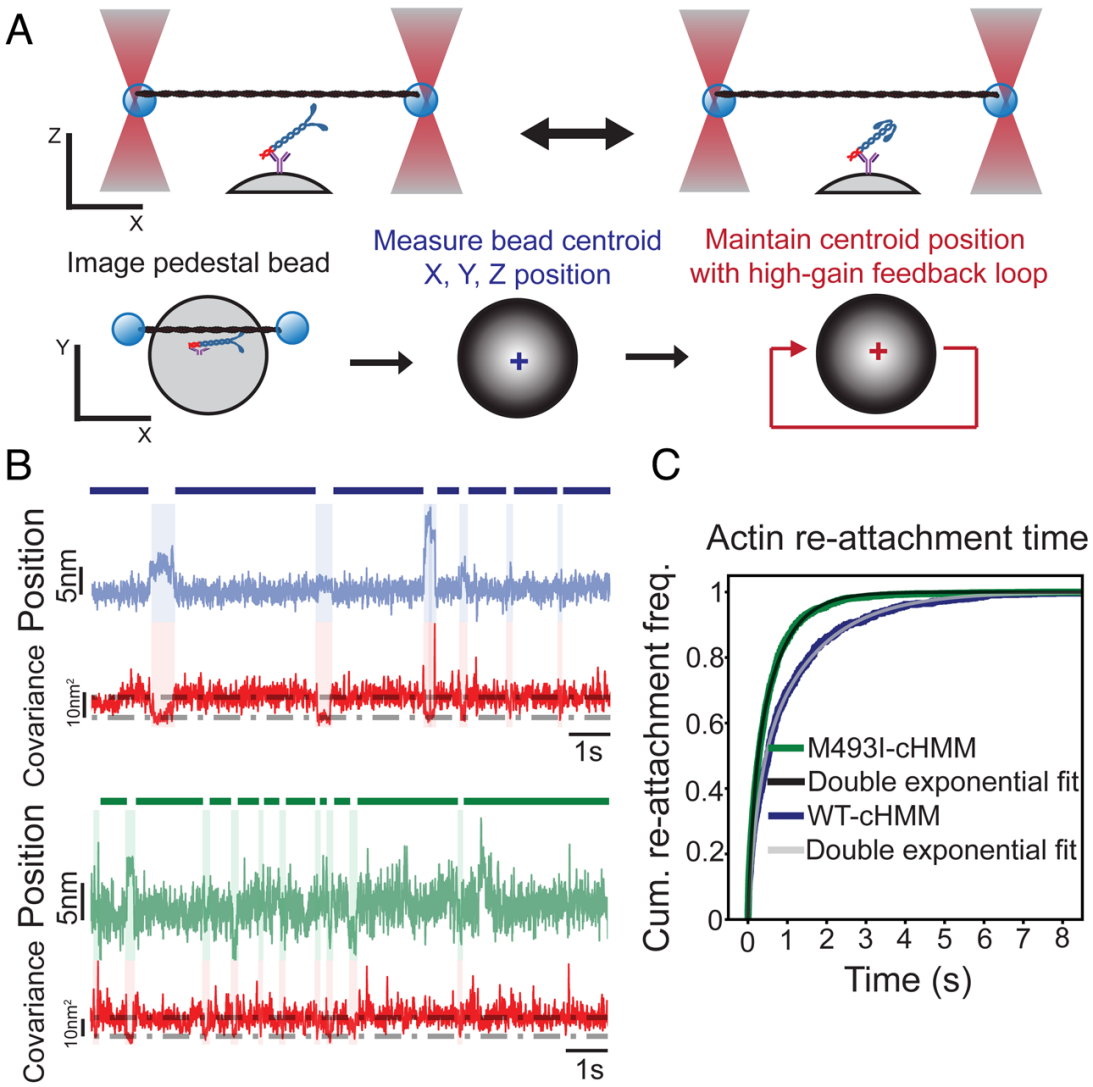
Actin reattachment rate measured by optical trap assays. (*A*) Schematic of myosin immobilization that preserves S1–S2 interactions for SRX formation, and high-gain feedback loop to stabilize the actin–myosin interaction zone. (*B*) Sample optical trap recordings showing shorter times between single actomyosin interactions for M493I myosin. (*C*) Cumulative distributions of reattachment times for M493I-cHMM and WT-cHMM showing faster reattachment rate for M493I myosin relative to WT.

The increased availability of DRX heads in M493I-cHMM is expected to increase interactions with actin filaments. To assess this possibility, we performed single-molecule actomyosin interaction assays at saturating (2 mM) ATP in our three-bead optical trap assay and measured the time between actomyosin interactions. In order to reliably measure the ON-rate of myosin to actin in the 3-bead assay, the technique was enhanced with a high-gain stage feedback system to maintain nanometer-scale precision in localizing the actin interaction zone (69) and careful optimization of each actin dumbbell for interaction with the myosin on the bead. Over the course of an acquisition of single actomyosin interactions, the actin filament position is centered and maintained relative to the cHMM (within the Brownian distribution of the optically trapped dumbbell), and the antibody-epitope adhesion scheme preserves the proximal S2 tail of the cHMM for forming SRX interactions (Fig. 5*A*).

The time from the end of one single-molecule actomyosin interaction and the start of the next was measured by a rise and then drop in bead covariance. In direct comparison, traces of WT-cHMM interactions showed a significantly slower reattachment to actin filaments (blue lines, Fig. 5*B*) than those of M493I-cHMM (green lines, Fig. 5*B*). Analysis of reattachment times across multiple molecules revealed a double-exponential distribution of attachment rates. Both WT-cHMM and M493I-cHMM shared a minor kinetic component comprising ~20% of the total amplitude, with similar rates (WT: 17 s^−1^, 95% CI: 5.5 to 36 s^−1^ and M493I: 23 s^−1^, 95% CI: 11 to 39 s^−1^). However, the dominant kinetic component, accounting for the majority of events, was nearly twice as fast in M493I-cHMM (1.62 s^−1^, 95% CI: 1.45 to 1.74 s^−1^) compared to WT-cHMM (0.83 s^−1^, 95% CI: 0.70 to 0.90 s^−1^, Fig. 5*C*). The fast phase may represent spurious detections or rapid reattachments without entering the SRX state, while the slow phase likely counts interactions that require a transition from SRX to DRX state. This suggests that the increased population of DRX heads in the M493I mutant enhances its ability to rapidly rebind actin.

### Discussion.

HCM is often described as a disease of hypercontractility, yet studies of individual purified myosin mutations have revealed mixed effects. Some mutations enhance motor activity, while others inhibit it. In this study, we characterized the mechanochemistry of a highly penetrant HCM mutation, M493I, and reconciled its seemingly contradictory kinetics. Although the mutation appears to reduce activity based on slower actin gliding motility, it ultimately leads to stronger and more sustained actin interactions: The mutant shows increased actin binding due to a twofold disruption in SRX state formation, prolonged attachment times driven by a fivefold slowing of ADP release, and elevated force production by actomyosin cross-bridges. These perturbations, in the context of a heterozygous myocardium, are likely to result in asynchronous contraction, aberrant TF activation, and septal stiffening hypertrophy, as seen in patients.

### A Relay Helix Point Mutation Disrupts the Kinetic Profile of Cardiac Myosin.

The relay helices of myosins are tightly conserved across the human genome; the position corresponding to M493 is one of the most variable residues in *Homo sapiens* myosin sequences but appears as a methionine in all class II myosins. In the transport myosins (classes V and VI), this position bears a valine or isoleucine, as in M493I. These myosins release ADP slowly, as their rate-limiting step, a key factor in their high DR and long actin attachment duration. In fact, the ADP release rate from AM•ADP of myosin V, at 11/s, is remarkably similar to the measured rate for M493I, and the DR of M493I-cS1 is much closer to that of myosin V than the low DR class II wild type myosin isoforms found in muscle (67). M493I is also rate-limited by ADP release, rather than P_i_ release, which is the rate-limiting step for WT cardiac myosin (50). In myosin IB, the exemplary force-sensing myosin, a threonine occupies this position (70). These differences in relay helix characteristic, with corresponding changes to myosin activity, point to the important allosteric effects of the relay helix in communicating between the nucleotide active site and the myosin lever arm. P_i_ release for WT cardiac myosin has been reported to occur in two phases for both HMM and S1 constructs, with the fast phase corresponding to the actin-stimulated structural transition to the strong-bound state and substep 1 of the power stroke. The relevance of the second phase is not clear, but it has been proposed as an on-actin ATP hydrolysis step (50, 51). It may also represent a relatively slow regulatory transition prior to actin interaction. The absence (or low detectability) of a slow phase for M493I-cHMM may mean that no such slow regulatory transition occurs, possibly because of enhanced affinity for the TF.

### Enhanced Attachment Duration Leads to High Loaded Force Production.

The tight regulation of molecular interactions underpinning the cross bridge cycling that powers cardiac function implies that dysfunction in diseases such as HCM results from a range of effects of mutations on myosin function that may lead to hypercontractility. Here, the presence of long actin attachment durations from slowed ADP release may be related to significantly enhanced force production by M493I myosin under hindering load. Our data suggest that the long-lived, high-force states in M493I myosin are primarily driven by slowed ADP release, which prolongs the lifetime of AM·ADP. The reduced distance parameter alone would not be expected to account for these effects, and enhanced force production under hindering load may be further reinforced by recruitment of the second myosin head. In the myocardium, this could lead to hypercontractility not only through increased force production by individual motors but also through the cooperative action of enhanced TF activation, inducing WT motors expressed from the other allele to contribute aberrantly high force. The apparent increase in actomyosin affinity for ADP is likely to shift the force–pCa relationship of M493I-bearing muscle fibers to the left, implying force production at lower [Ca^2+^], which would slow relaxation rates and compromise diastole. These effects may contribute to significant stiffening of the myocardium and imbalances in forces between neighboring myocytes, leading to asynchronous contraction, disorder, and interstitial fibrosis.

### Disruptions to the SRX State Enhance Actin Interactions.

HCM mutations do not universally alter the SRX-DRX equilibrium of purified, soluble myosin. Previous work from our group has found, for instance, that the converter domain mutation R712L has no effect on SRX-DRX equilibrium, while E497D (also found in the relay helix) increases the equilibrium constant of the SRX-DRX equilibrium, evidence for destabilization of the SRX myosin heads (48). Thus, the hypothesis that an increase in available DRX heads contributes to HCM is not universally true for soluble myosin. The SRX-DRX transition may be different in a native thick filament, so additional experiments need to be performed in situ. The mutation M493I nearly doubles the equilibrium constant of the SRX/DRX transition, consistent with the head availability model of HCM. This increase in the equilibrium constant of the SRX/DRX transition changes the percentage of DRX heads from 25 to 35%; if this change is present in cardiomyocytes bearing the M493I mutation, such an increase in heads in the interfilament lattice would significantly affect TF activation, synchronous force generation, and diastole. This mutation also doubles *V_max_* of actin-activated ATP hydrolysis measured in bulk kinetics, as expected with more available motor domains, and doubles the actin reattachment rate measured in single-molecule optical trap assays. With the compounding impacts of disruption to the SRX “off” state on two-headed myosins with higher DR than WT, small effects on mechanical and kinetic transitions are amplified to create significant composite changes to myosin function.

## Materials and Methods

### Protein Purification.

HMM of human β-cardiac myosin (MYH7) was expressed in mouse C2C12 myoblasts and purified according to previously established methods (16, 27, 48, 71). Briefly, the cHMM cDNA was cloned into the pShuttle-IRES-hrGFP-1 vector (Agilent Tech., Santa Clara, CA). The AdcHMM-Flag virus was prepared and amplified for expression of cHMM protein in C2C12 cells. For the cHMM S2Tag construct, the sequence of the epitope (AEKHRADLSRE) was introduced into the coiled-coil S2 domain of β-cHMM, followed by two additional heptads of cardiac S2 sequence and a FLAG tag at the C-terminus (44, 72). The associated essential and regulatory light chains are mouse endogenous genes Myl1 and Mylpf expressed in C2C12 myotubes (73). Mutant adenovirus constructs were synthesized by Genewiz (South Plainfield, NJ). The virus was expanded by infection of a large number of plates of confluent Ad293 cells at multiplicity of infection (MOI) of 3 to 5. The virus was harvested from the cells and purified by CsCl density sedimentation yielding final virus titers of 10^10^–10^11^ plaque forming units per mL (pfu mL^−1^). Confluent C2C12 myoblasts were infected with replication defective recombinant adenovirus (AdcHMM-Flag) at 2.7 × 10^8^ pfu mL^−1^ in fusion medium (89% DMEM, 10% horse serum, 1% FBS). Expression of recombinant cHMM was monitored by accumulation of coexpressed GFP fluorescence in infected cells. Myocyte differentiation and GFP accumulation were monitored for 216 to 264 h after which the cells were harvested. Cells were chilled, media removed, and the cell layer was rinsed with cold PBS. The cell layer was scraped into Triton extraction buffer: 100 mM NaCl, 0.5% Triton X-100, 10 mM Imidazole pH 7.0, 1 mM DTT, 5 mM MgATP, and protease inhibitor cocktail (Sigma, St. Louis, MO). The cell suspension was collected in an ice-cold Dounce homogenizer and lysed with 15 strokes of the tight pestle. The cell debris in the whole cell lysate was pelleted by centrifugation at 17,000 × g for 15 min at 4 °C. The Triton soluble extract was fractionated by ammonium sulfate precipitation using sequential steps of 0 to 30% and 30 to 60% saturation. The cHMM precipitates between 30% and 60% saturation of ammonium sulfate. The recovered pellet was dissolved in and dialyzed against 10 mM Imidazole, 150 mM NaCl, pH 7.4 for affinity purification of the FLAG-tagged cHMM on M2 mAb-Sepharose beads (Sigma). Bound cHMM was eluted with 0.1 mg/mL FLAG peptide (Sigma). Protein was concentrated and buffer exchanged on Amicon Ultracel-10 K centrifugal filters (Millipore; Darmstadt, Germany), dialyzed exhaustively into 10 mM MOPS, 100 mM KCl, 1 mM DTT before a final centrifugation at 300,000 × g for 10 min at 4 °C. Aliquots were drop frozen in liquid nitrogen and stored in vapor phase at –147 °C. Purified WT human β-cHMM and M493I HCM variants were routinely analyzed by SDS-PAGE.

Actin was purified from rabbit skeletal muscle (74). Native porcine cardiac TFs were prepared according to the procedure of Spiess et al. (75) as modified by Matsumoto et al. (76).

### Protein Gels.

Proteins were prepared in 1× Laemmli buffer with 0.5% DTT and run on NuPAGE Bis-Tris acrylamide gels 4 to 12% (Invitrogen), followed by Coomassie staining and destaining according to standard procedures. Gels were imaged on a Licor Odyssey gel imaging dock; all gel images are unedited and uncropped except to exclude irrelevant lanes.

### In Vitro Gliding Assays.

Measurement of in vitro motility of human β-cHMM2.0 was done as previously described for skeletal muscle myosin (16, 27, 76–78). Nitrocellulose-coated glass coverslips were incubated with 0.15 mg/mL of the S2Tag mAb 10F12.3, followed by blocking the surface with 1% BSA. β-cHMM proteins were diluted in motility buffer (MB) (25 mM imidazole, pH 7.8, 25 mM KCl, 4 mM MgCl_2_, 1 mM MgATP, 1 mM DTT) supplemented with 1% BSA (MB/BSA) to a final concentration of 10 μg/mL. The antibody-coated coverslips were incubated with β-cHMM2.0 for ∼2 h in a humidified chamber at 4 °C. The coverslips were washed with MB/BSA, followed by actin blocking with 1 μM F-actin, and washes with motility buffer, then transferred to a 15-μL drop of 2 nM rhodamine-phalloidin–labeled actin in a modified motility buffer (with 7.6 mM MgATP, 50 mM DTT, 0.5% methyl cellulose, 0.1 mg/mL glucose oxidase, 0.018 mg/mL catalase, 2.3 mg/mL glucose) in a small parafilm ring fixed on an alumina slide with vacuum grease. The chamber was observed with a temperature-controlled stage and objective set at 32 °C on an upright microscope with an image-intensified charge-coupled device camera capturing data to an acquisition computer at 30 Hz. Movement of actin filaments from 500 to 1,000 frames of continuous imaging was analyzed with semiautomated filament tracking programs as previously described (76). The trajectory of every filament with a lifetime of at least 10 frames was determined; the instantaneous velocity of the filament moving along the trajectory, the filament length, the distance of continuous motion and the duration of pauses were tabulated. A weighted probability of the actin filament velocity for hundreds of events was fit to a Gaussian distribution and reported as a mean velocity and SD for each experimental condition. For presentation purposes, background was subtracted using a rolling ball of 1 pixel radius with sliding paraboloid shape, followed by Gaussian smoothing of size 3 × 3 pixels, both in ImageJ. Color-coded time projections were produced with the ImageJ plugin ColorCodingFrames.ijm (https://github.com/hansenjn/ColorStackByTimeAndProject).

### Three-Bead Optical Trap Assay.

Optical trap assays were performed at room temperature (20 °C) in flow cell chambers constructed of a microscope slide (75 × 25 mm) with coverslip (40 × 25 mm) adhered by double-sided tape. The coverslip was coated with 0.1% nitrocellulose (EMS) in amyl acetate (EMS) including dilute 2.5 μm silica pedestal beads (Polysciences). All reagents were in Assay Buffer (AB) which contained 60 mM MOPS, 25 mM KCl, 1 mM DTT, 1 mM MgCl_2_, and 1 mM EGTA unless otherwise specified. Antibodies to the S2Tag were diluted to 0.03 mg/mL and incubated for 60 s to adhere to the coverslip surface. The chamber was then blocked 2× for 3 min each in 1 mg/mL BSA, then incubated in cHMM (WT or M493I) diluted to 0.05 to 0.1 μg/mL in myosin buffer (AB except with 300 mM KCl) for 3 min, then blocked 2× for 2 min each in 1 mg/mL BSA. The experimental solution was added to the flow cell containing 0.2 nM actin filaments containing 10 to 15% biotinylated actin and 85 to 90% rabbit skeletal muscle actin, which was stabilized by equimolar rhodamine-labeled phalloidin, as well as an oxygen scavenging system plus glucose [100× GOC was prepared by dissolving 7.5 mg of glucose oxidase (Sigma-Aldrich G2133) in 200 μL of 10 mM HEPES pH 7.4, and adding 60 μL of bovine liver catalase (Sigma-Aldrich C3155)], spinning at 17,900×g for 5 min, and filtering through 0.22 μm filter unit (EMD Millipore SLGV004SL), and varying concentrations of MgATP. Finally, 750 nm polystyrene beads, prepared by incubating 0.4 ng of beads with 10 μL of 10 mg/mL neutravidin solution in water overnight at 4 °C with rotation before washing 2× with AB+MgATP were diluted 1:100 in AB+MgATP. 3 μL of the bead suspension was added to the chamber before it was sealed with vacuum grease. Chambers were imaged for <60 min after sealing. Concentrations of antibody and myosin were optimized such that one of each 5 to 10 locations tested showed actomyosin interactions. Chambers constructed with no antibody in the first flow step, but following all other steps the same, showed no actomyosin interactions.

Experiments were performed on a dual-beam optical trap setup constructed on a Nikon Eclipse TE2000 microscope. A 1064-nm laser was split into two by polarization, with each beam passing through a 1-D electro-optical deflector, each of which can deflect the beam based on voltage. The position of one trap was also varied by rotating a motorized mirror conjugate to the back focal plane of the objective. The beams were focused at the focal plane with a Nikon Plan Apo 60× water immersion objective (1.2 NA), and the traps were projected by a Nikon HNA oil condenser (1.4 NA) onto two quadrant photodiodes (JQ-50P, Electro Optical Components Inc.) for position and force detection. Data acquisition, feedback, and beam position were controlled with custom-built virtual instruments programmed on a LabVIEW multifunction I/O device with a built-in FPGA (PXI-7851); data were acquired at 250 kHz. Beads were caught by moving the microscope stage to steer them into the traps. Stiffness of the traps were 0.07 to 0.1 pN/nm, as calculated via the power spectrum of the beads’ fluctuations. An actin filament 5 to 10 μm long was then suspended between the two beads, and the position of one trap was moved away from the other with the motorized mirror conjugate until 4.5 to 5.5 pN of pretension was applied to the actin filament. Pedestal beads were tested by pressing the actin filament against the pedestal to detect bead variance changes. Once actomyosin interactions were detected, a piezo-electric stage (Mad City Labs) was used to optimize the interaction zone, by visualizing both number (for high [ATP]) and bead displacement (for low [ATP]) of attachment events.

A stage feedback system imaged the pedestal bead with a monochrome camera (Thorlabs) as described previously (69). The gain of the feedback loop was set to maintain <3 nm error in X and Y positions, and <10 nm error in Z position. Upon engagement of the feedback loop, data were acquired without adjustment to bead positions.

Isometric feedback experiments were conducted using a digital feedback loop and the EODs to steer the beam position (62). A feedback loop held the position of the pointed-end associated bead (referred to as the transducer) constant by modulating the position of the trap holding barbed-end associated bead, termed the motor trap. Since the actin filament is between the two beads inside the feedback loop, its position was maintained continuously, allowing the myosin to develop isometric force during its interaction with actin. The response time of the feedback loop during myosin interactions was ~10 ms. The average force during an interaction was calculated by averaging the force on the motor bead starting 2 ms after detected attachment through 2 ms before detachment. The baseline force on the motor bead 4 ms after detachment was subtracted from this average force.

### Optical Trap Data Analysis.

Optical trap data were analyzed using python scripts available at GitHub.com/bobcail. First, the running covariance of the beads from a 15 s recording was calculated over an 8 ms sliding window and fit by a double Gaussian distribution, with high-covariance values corresponding to unbound actin and low-covariance values corresponding to actomyosin interaction events. Events were selected by finding when the covariance dropped below the high peak, then below the lower peak, then rose above the high peak again. Events shorter than 16 ms (the dead time of the instrument) were excluded. The duration of each event was calculated from these trigger points; event durations were fit by single exponential functions using MEMLET (79). Substep sizes were calculated from 1 μM ATP data by averaging 1 ms of the bead position at three points: 1) 4 ms prior to attachment, 2) 2 ms after attachment, and 3) 4 ms before detachment. Substep 1 was calculated as 2-1, and substep 2 was calculated as 3 to 2. Ensemble averaging was performed by aligning all events to the start (forward average) or stop (reverse average), and events were extended to the longest event time by averaging 4 ms of data, located 1 ms from the covariance transition point. Events were averaged with each event contributing equal weight to the ensemble. Ensembles were fit by single exponential functions by nonlinear least squares fitting in python. All plots were generated in python.

### Stopped-Flow Experiments.

Stopped flow experiments were conducted in an SX20 Stopped Flow Spectrometer (Applied Photophysics), with fluorescence excitation provided by a 100-W Hg lamp with a monochromator. Data were acquired and analyzed using Pro Data-SX software. Pyrene-actin was produced following previously published protocols (49). Pyrene-actin for ADP release and ATP-induced actomyosin dissociation was excited at 365 nm, and the fluorescence emission peak was detected through a 400 nm long-pass filter. For ADP release, 1 μM M493I or 200 nM WT myosin heads (incubated with 0.01 U/mL apyrase on ice for 30 min) was mixed with equimolar pyrene-actin with 280 μM ADP for 10 min at RT in KMg25 buffer [60 mM 3-(N-morpholino)propanesulfonic acid, pH 7.0, 1 mM MgCl_2_, 1 mM EGTA, and 1 mM DTT], then mixed with 4 mM ATP in KMg25 and fluorescence intensity was observed for 1 s. Final concentrations in cuvette: 0.5 μM heads, 0.5 μM pyrene-actin, 140 μM ADP, 2 mM ATP for M493I, 0.1 μM heads, 0.1 μM pyrene-actin, 140 μM ADP, 2 mM ATP for WT. For ATP binding, 1 μM myosin heads (incubated with 0.01 U/mL apyrase on ice for 30 min) were mixed with 1 μM pyrene-actin for 10 min at RT in KMg25 buffer (60 mM MOPS pH 7, 25 mM KCl, 1 mM EGTA, 1 mM MgCl_2_, 1 mM DTT), then mixed with 0 to 1,200 μM ATP in KMg25 and fluorescence intensity was observed for 1 s. Final concentrations in cuvette: 0.5 μM heads, 0.5 μM pyrene-actin, 0 to 600 μM ATP. For actin-activated phosphate release, fluorescently labeled mutant phosphate binding protein (MDCC-labeled PiBiP; (7-diethylamino-3-((((2-maleimidyl)ethyl)amino)carbonyl) coumarin)-labeled phosphate binding protein) was excited at 430 nm, and fluorescence was detected with a 440 nm long-pass filter. The instrument was precleaned with a phosphate mop consisting of 0.3 mM 7-methylguanosine and 0.1 U/mL bacterial nucleoside phosphorylase in phosphate release buffer (TF-calcium buffer, 0.11 mM CaCl_2_, 10 mM KCL, 2 mM MgCl_2_, 10 mM MOPS pH 7.2). Phosphate release was measured in phosphate release buffer with 5 μM MDCC-labeled PiBiP, 0.1 mM 7-methylguanosine, and 0.01 U/mL bacterial nucleoside phosphorylase; 4 μM myosin heads were mixed with 4.1 μM ATP in the preincubation loop for 10 s, followed by mixing with 60 μM TFs, and fluorescence measured for 21 s on a split time base (1,000 points in first second, 1,000 points for 20 s). Final concentrations in cuvette: 1 μM myosin heads, 1.025 μM ATP, 30 μM TFs.

For single-nucleotide turnover, 200 nM myosin heads were incubated with 220 nM MantATP in the preincubation loop for 10 s, followed by mixing with 2 mM unlabeled ATP. Mant-ATP was excited by FRET from tryptophan 508 with 295 nm excitation, and fluorescence was collected with a 400 nm long-pass filter. Final concentration in cuvette: 50 nM myosin heads, 50 nM MantATP, 1 mM unlabeled ATP. For proteolytic fragment formation, 0.5 mg/mL HMM (apyrase treated at 0.1 U/mL on ice for 30 min before use) was incubated in a total of 60 μL KMg25 with 5 mM cysteine and 18.1 μM papain (diluted from 1.09 mM stock, Sigma-Aldrich p3125) for 5 min for WT or 3 min for M493I at room temperature. The reaction was quenched with addition of 25 μM E-64 protease inhibitor (Cayman Chemical), and 10 μL was reserved for SDS-PAGE analysis; in parallel, a sample was prepared identically but without the addition of papain, to test samples and the effect of incubation times and the E-64. Samples were incubated on ice until single-nucleotide turnover, which was performed identically as for untreated samples. One data value for M493I papain-treated single turnover was 5 SD away from the mean of the others and was excluded. Stopped-flow data were fitted by single exponentials and rates were fitted where appropriate by the Michaelis–Menten equation using custom Python scripts.

### Actin-Activated ATP Hydrolysis.

Steady-state ATPase experiments were measured spectrophotometrically (Agilent) using standard protocols (49). Briefly, an assay buffer (TF-calcium buffer) containing 0.11 mM CaCl_2_, 10 mM KCL, 2 mM MgCl_2_, 10 mM MOPS pH 7.2, 2 mM MgATP, 0.2 mM NADH, 20 U/mL lactate dehydrogenase, 100 U/mL pyruvate kinase, and 0.5 mM phospho(enol)pyruvate was incubated with 100 nM myosin heads 0 to 60 μM TFs (all final concentration) and the absorbance was measured at 340 nM over 110 s. The slope of the fluorescence change was fitted by a straight line, the slope giving the rate of decrease in NADH concentration (extinction coefficient of 6,220 M^−1^ cm^−1^) which corresponds to the rate of ATP hydrolysis. Per-head ATPase rates were fitted to the Michaelis–Menten equation with custom python scripts.

### Statistical Tests.

Purified WT and M493I-cHMM preparations represent three biological replicates. All measurements include three technical replicates. Measured values are reported as mean ± SD. unless otherwise stated. Fitted values are reported as value with 95% CI as determined by bootstrapping for 1,000 iterations. Unpaired Welch’s *t* tests were performed in Python to measure statistical significance. Data were assumed to be normally distributed, but no formal normality test was performed.

## Supplementary Material

Appendix 01 (PDF)

Movie S1. WT actin gliding motility assay. Rhodamine-phalloidin labelled actin filaments are propelled by WT-cHMM molecules adhered to the coverslip at a total motor concentration of 10 μg/mL. Scale bar: 10 μm.

Movie S2. M493I actin gliding motility assay. Rhodamine-phalloidin labelled actin filaments are propelled by M493I-cHMM molecules adhered to the coverslip at a total motor concentration of 10 μg/mL. Scale bar: 10 μm.

Movie S3. Mixed motor actin gliding motility assay. Rhodamine-phalloidin labelled actin filaments are propelled by a mixture of 50% WT-cHMM molecules and 50% M493I-cHMM molecules adhered to the coverslip at a total motor concentration of 10 μg/mL. Scale bar: 10 μm.

## Data Availability

Representative traces of kinetics and optical trap acquisitions, representative micrographs of fluorescence microscopy data, and unedited SDS-PAGE/Coomassie gels are presented in this manuscript; all fits and statistics are reported, and all data are included. Raw data are available at https://zenodo.org/records/17477924 (80). Analysis code is available at GitHub.com/bobcail. All other data are included in the manuscript and/or supporting information.
