# Supplementary material for: A myosin hypertrophic cardiomyopathy mutation disrupts the super-relaxed state and boosts contractility by enhanced actin attachment
Source: Proc Natl Acad Sci U S A. 2025 Dec 24;122(52):e2521561122. doi: 10.1073/pnas.2521561122 (PMC12772213; doi:10.1073/pnas.2521561122)
Supplement: Supplementary file 1 — Appendix 01 (PDF) [file pnas.2521561122.sapp.pdf]

Supplemental file for:

A myosin hypertrophic cardiomyopathy mutation disrupts the super-relaxed state and boosts contractility by enhanced actin attachment

Authors: Robert C. Cail[1,2], Bipasha Barua[3], Faviolla A. Báez-Cruz[1], Donald A. Winkelmann[3], Yale E. Goldman[1,2,4*], and E. Michael Ostap[1,2*]

[1]Department of Physiology, Perelman School of Medicine, University of Pennsylvania

[2]Pennsylvania Muscle Institute, Perelman School of Medicine, University of Pennsylvania [3]Department of Pathology and Laboratory Medicine, Robert Wood Johnson Medical School, Rutgers University

[4]Department of Pharmacology and Department of Molecular and Cell Biology, University of California, Davis

*Correspondence to E. Michael Ostap: ostap@pennmedicine.upenn.edu

*or Yale E. Goldman: yegoldman@ucdavis.edu

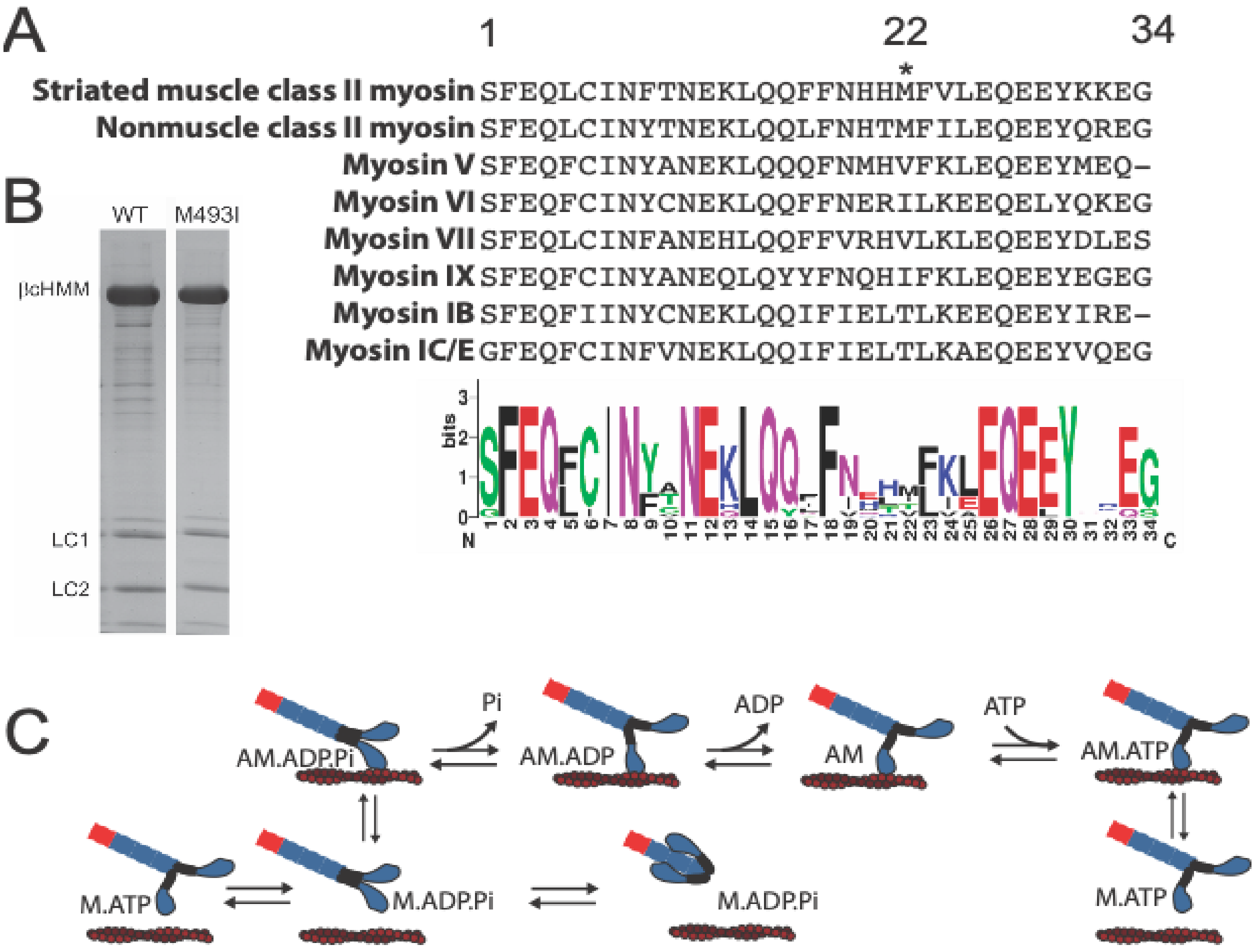

Fig S1: WT and M493I myosins. A) Comparison of relay helix sequences across many *H. sapiens* myosin paralogs. M493, at position 22 in the relay helix, is conserved in class II myosins but variable across paralogs. B) SDS-PAGE gels of purified WT- and M493I-cHMMs expressed recombinantly in mouse C2C12 myoblasts and purified. C) Scheme 1: ATPase scheme of class II myosins, which includes the SRX-DRX regulatory transition for M.ADP.Pi.

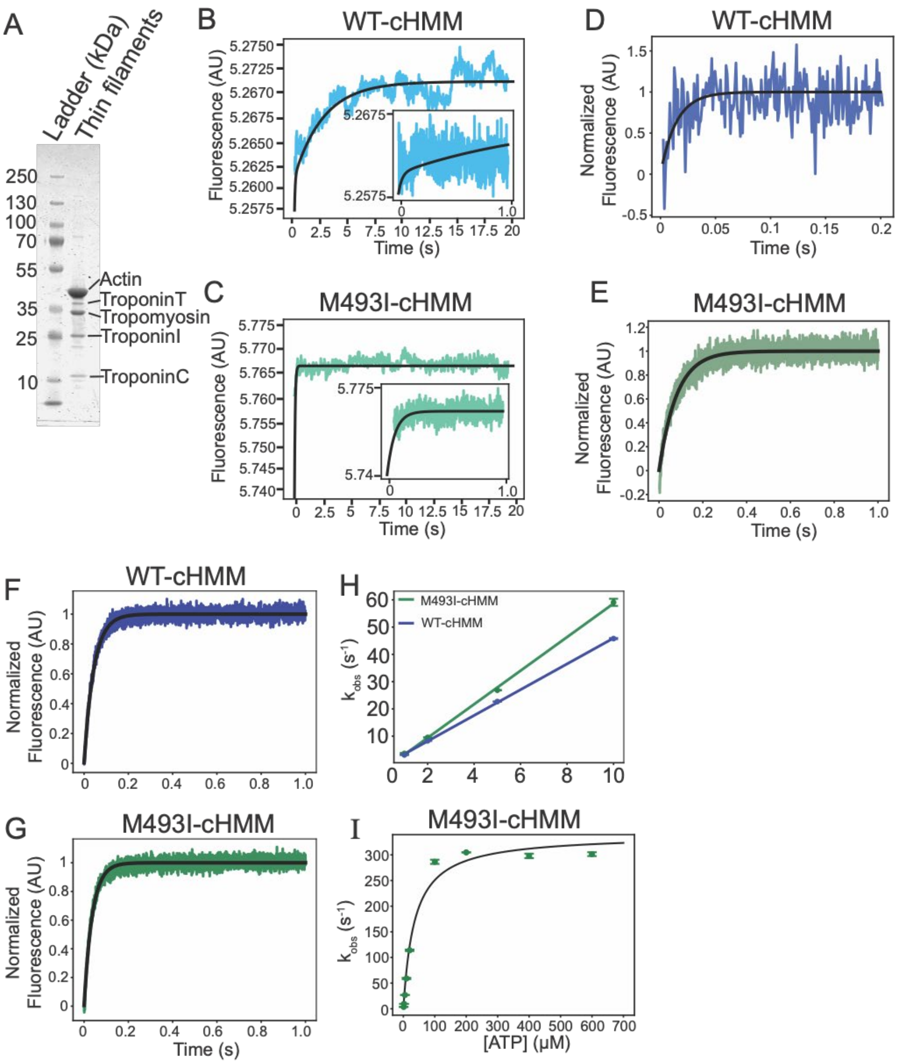

Fig S2: Transient kinetics of WT- and M493I-cHMMs. A) SDS-PAGE of porcine ventricular thin filaments (TFs). B-C) Example phosphate release transient of WT (B) with double exponential fitted curve and M493I (C, single exponential fit) sampled at 50 Hz. Insets: First 1 second of each trace, sampled at 250 Hz, with double exponential (WT) or single exponential (M493I) fits. D-E) Example transients of ADP release for WT (D) and M493I (E) from pyrene-labeled actin. F-G) Example transients of ATP binding for WT (F) and M493I (G) at 5 μM ATP H) k_{obs} for myosin dissociation by pyrene fluorescence change at low ATP concentrations to compare apparent 2nd-order rate constant of ATP binding for M493I (green) *vs*, WT (blue) cHMM. Mean +/- S.D. as error bars. I) Michaelis-Menten curve fitted to dissociation rate constant *vs*. [ATP] for M493I.

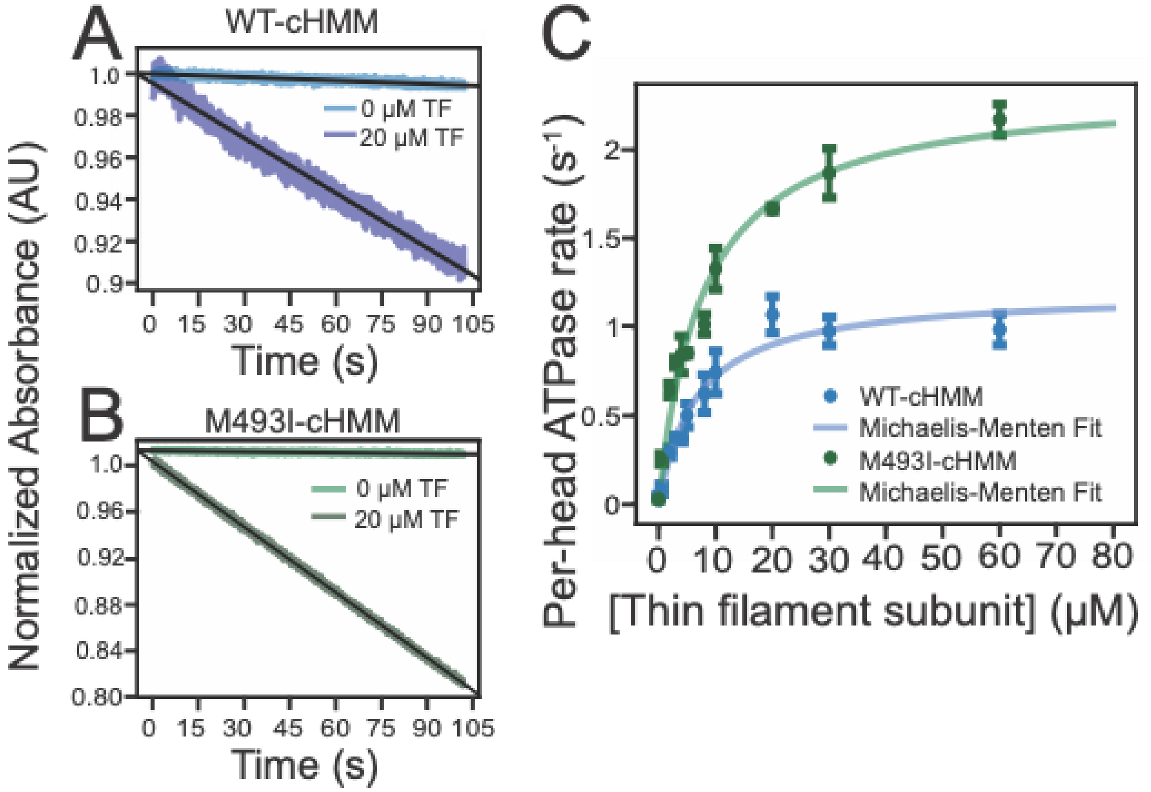

Fig S3: Actin-activated steady-state ATPase activity. A-B) Sample NADH absorbance traces from WT (A) and M493I (B) demonstrating linear decrease in the absence and presence of 20 mM TF subunit concentration. C) ATPase rate per head *vs*. [TF] with fitted Michaelis-Menten curves demonstrating approximate doubling of steady-state V_{max} for M493I myosin relative to WT.

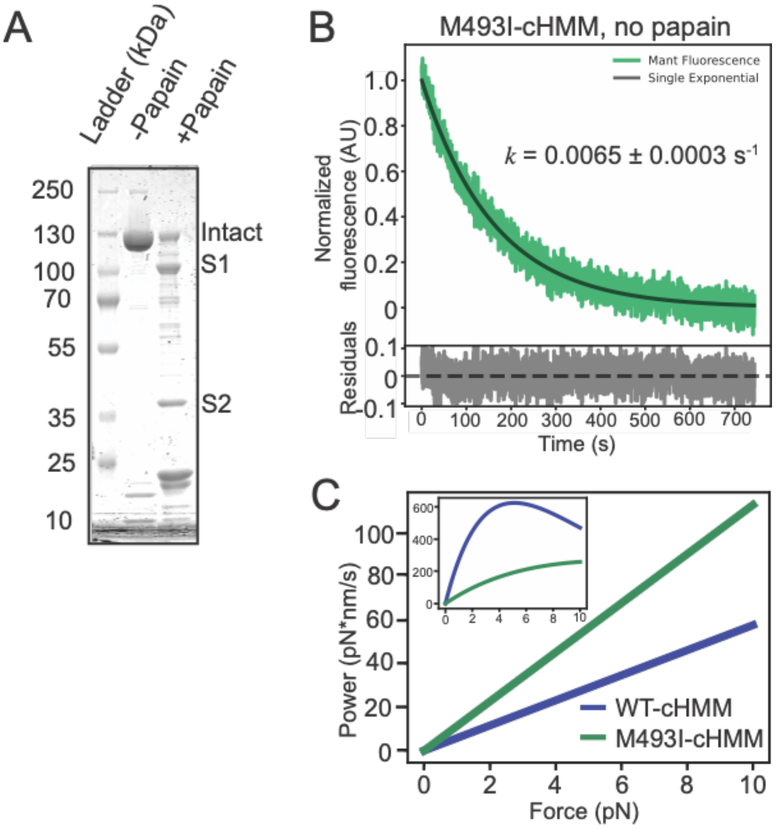

Fig S4: Papain digestion. A) SDS-PAGE of M493I myosin demonstrating cleavage into S1, S2, and limited intact myosin in the papain digest. B) Mant nucleotide fluorescence transient of an undigested M493I control using digestion buffer with E-64 papain inhibitor is not significantly different from that of M493I protein not exposed to the digestion conditions (Fig 4A). C) Duty-ratio corrected power output for WT-cHMM and M493I-cHMM calculated over a range of forces by multiplying the uncorrected power (eq. 4) by the force-dependent duty ratio (eq. 3)

Inset: Estimated power output without accounting for the load-dependent duty ratio.

Video S1: WT actin gliding motility assay. Rhodamine-phalloidin labelled actin filaments are propelled by WT-cHMM molecules adhered to the coverslip at a total motor concentration of 10 μg/mL. Scale bar: 10 μm.

Video S2: M493I actin gliding motility assay. Rhodamine-phalloidin labelled actin filaments are propelled by M493I-cHMM molecules adhered to the coverslip at a total motor concentration of 10 μg/mL. Scale bar: 10 μm.

Video S3: Mixed motor actin gliding motility assay. Rhodamine-phalloidin labelled actin filaments are propelled by a mixture of 50% WT-cHMM molecules and 50% M493I-cHMM molecules adhered to the coverslip at a total motor concentration of 10 μg/mL. Scale bar: 10 μm.
